# Opioid, antipsychotic and hypnotic use in end of life in long-term care facilities in six European countries: results of PACE

**DOI:** 10.1093/eurpub/cky196

**Published:** 2018-10-04

**Authors:** Marc Tanghe, Nele Van Den Noortgate, Lara Pivodic, Luc Deliens, Bregje Onwuteaka-Philipsen, Katarzyna Szczerbińska, Harriet Finne-Soveri, Danni Collingridge-Moore, Giovanni Gambassi, Lieve Van den Block, Ruth Piers, PACE

**Affiliations:** 1 End-of-Life Care Research Group, Ghent University, Ghent, Belgium; 2 Department of Geriatrics, Ghent University Hospital, Ghent, Belgium; 3 End-of-Life Care Research Group, Vrije Universiteit Brussel, Brussels, Belgium; 4 Medisch Centrum, Vrije Universiteit, Amsterdam, The Netherlands; 5 Jagiellonian University Medical College, Kraków, Poland; 6 National Institute for Health and Welfare, Helsinki, Finland; 7 Lancaster University, Lancaster, UK; 8 Fondazione Policlinico Universitario A. Gemelli, IRCCS and Università Cattolica del Sacro Cuore, Roma, Italy

## Abstract

**Background:**

Opioids, antipsychotics and hypnotics are recommended for comfort care in dying. We studied their prescription during the last 3 days in residents deceased in the long-term care facility (LTCF).

**Methods:**

In a retrospective, cross-sectional survey in Belgium, England, Finland, Italy, the Netherlands and Poland, LTCFs, selected by proportional stratified random sampling, reported all deaths over the previous 3 months. The nurse most involved in the residents’ care reviewed the chart for opioid, antipsychotic and hypnotic prescription, cause of death and comorbidities. Multivariable logistic regression was performed to adjust for resident characteristics.

**Results:**

Response rate was 81.6%. We included 1079 deceased residents in 322 LCTFs. Opioid prescription ranged from 18.5% (95% CI: 13.0–25.8) of residents in Poland to 77.9% (95% CI: 69.5–84.5) in the Netherlands, antipsychotic prescription from 4.8% (95% CI: 2.4–9.1) in Finland to 22.4% (95% CI: 14.7–32.4) in Italy, hypnotic prescription from 7.8% (95% CI: 4.6–12.8) in Finland to 47.9% (95% CI: 38.5–57.3) in the Netherlands. Differences in opioid, antipsychotic and hypnotic prescription between countries remained significant (*P* < 0.001) when controlling for age, gender, length of stay, cognitive status, cause of death in multilevel, multivariable analyses. Dying from cancer showed higher odds for receiving opioids (OR 3.51; *P* < 0.001) and hypnotics (OR 2.10; *P* = 0.010).

**Conclusions:**

Opioid, antipsychotic and hypnotic prescription in the dying phase differed significantly between six European countries. Further research should determine the appropriateness of their prescription and refine guidelines especially for LTCF residents dying of non-cancer diseases.

## Introduction

Long-term care facility (LTCF) residents in Europe evolve to a highly dependent population with complex, often incurable multi-morbidity.^1^ Consequently, palliative and terminal care should be key components in LTCF care, with adequate pain- and symptom-management as a priority.

Previous research documented a high prevalence of pain in LTCF residents. In a cross-sectional study in three European countries, the presence of pain varied between 32% and 57%. In nearly half of the cases, pain was present every day and in over 50%, pain was rated moderate-to-severe.^2^ A longitudinal study in the Netherlands revealed pain prevalence up to 68%, with 41% of residents in persistent pain.^3^ With regard to other symptoms, this study indicated that agitation is the most common symptom, with prevalence ranging from 57% to 71%.^3^

Pain treatment in a LTCFs is evolving, illustrated by an increase in opioid prescription in LTCFs.^4^ Nevertheless, recent research established undertreatment in residents with persistent pain.^5^ Especially the group of residents with cognitive impairment, received less opioid analgesics.^6^ In contrast, residents with dementia received more psychotropic medication,^7^ although their use is recently decreasing in the long-term care.^4^^,^^8^ Besides these studies about central nervous agents in general, specific data about antipsychotic and hypnotic prescription in LTCF residents near to death are rare as some studies on medication use in LTCFs exclude dying patients.^2^^,^^4–7^

In the last days of life, symptoms evolve rapidly. Sleep disturbance, agitation and neuropsychiatric symptoms decrease, while pain, feeding problems, breathing abnormalities, apathy and anxiety increase.^9^ Pain prevalence up to 78%^3^ has been reported.

Recent guidelines concerning terminal care recommend the use of opioids, hypnotics and antipsychotics to control pain, dyspnoea, agitation, anxiety and delirium^10^ in the dying phase.

Existing studies examining the impact of the guidelines regarding medication prescription at the end of life often focused on specific populations such as cancer patients and patients with dementia or are performed in acute care hospitals and palliative care settings. Consequently, to date, little is known about the prescription of opioids, hypnotics and antipsychotics in the last days of life in a general LTCF population. The PACE study (PAlliative Care for the Elderly), an EU-funded research project to assess quality of palliative care delivery in the European community‘s LTCFs, created the opportunity to conduct research in a larger, European population sample and allowed epidemiological comparison of the factual practice between participating countries. In this study, following research questions were addressed: (i) what is the prevalence of the prescription of opioids, antipsychotics and hypnotics in the last 3 days of life in LTCFs’ residents in six European countries and (ii) what factors are associated with this medication prescription?

## Methods

### Study design, setting and participants

In six participating countries, Belgium, England, Finland, Italy, the Netherlands and Poland a cross-sectional survey collected data on deceased LTCF residents. Countries were selected in order to obtain a good spread in geography, history of economic growth, healthcare system and level of palliative care development. The study methods are described in the published study protocol.^11^ In this article, ‘LTCF’ refers to a ‘collective institutional settings where care, on-site provision of personal assistance of daily living, and on-site or off-site provision of nursing and medical care, is provided for older people who live there, 24 hours a day, 7 days a week, for an undefined period of time’^,^. LTCFs were identified using proportional stratified random sampling, to guarantee nation-wide representativeness. Participating LTCFs reported all residents who died in a retrospective 3-month period, prior to the researchers visit to the LTCF. For this survey, we included residents who died in their LTCF and of whom the nurses’ questionnaire was completed.

### Data collection

Through an anonymized procedure, structured after-death questionnaires, regarding each deceased resident were sent to the treating physician, the nurse or care assistant most involved in the resident’s care and the LTCF management.

### Measurements

Demographic data and length of stay were extracted from the LTCF managements questionnaire regarding the deceased resident; LTCF characteristics from the LTCF managements questionnaire regarding their LTCF. In this study, ‘LTCF type’ refers to the staffing structure, depending on whether physicians and nurses are on-site or off-site. In every participating country, we found LTCFs with nurses on site 24/7, and physicians off-site. In Italy, the Netherlands and Poland, some LTCFs reported physicians and nurses on-site. LTCFs with on-site care assistants and off-site nurses and physicians only participated in the study in England.^11^

Based on the nursing records, the nurses provided information on the residents prescriptions, functional and cognitive status, dementia status and cause of death. Based on a list of available medications per country, the nurses executed a chart review to check whether or not opioids (e.g. morphine, oxycodone, hydromorphone, fentanyl, buprenorphine, tramadol), antipsychotics (e.g. haloperidol, risperdone, olanzapine, clotiapine) and hypnotics (e.g. midazolam, oxazepam, lorazepam, lormetazepam, zopiclone, zolpidem, zaleplon) had been prescribed to the resident in the last 72 hours of life. Functional and cognitive status was estimated by the Bedford Alzheimer Nursing Severity scale (BANS-S), a rating scale, comprising cognitive and functional items, developed for grading severity of dementia.^12^ Higher scores indicate higher functional disability and dependency. In the database, a resident ‘with dementia’ was defined as a resident to whom the nurse and/or the physician referred to as a resident with dementia. Cause of death was determined by means of a predefined checklist. The list of questions of the nurses' PACE-questionnaire, used in this paper (see Supplementary appendix).

### Statistical analysis

Descriptive statistics were provided per country as percentages (for categorical outcomes) and mean and SD (for continuous outcomes). Differences in residents’ and LTCFs’ characteristics between countries were explored by means of normal, multinomial and logistic regression, depending if the dependent variable was continuous, categorical or binary. Second, the estimated percentage and corresponding 95% CI of opioid, antipsychotic and hypnotic prescription was estimated using a mixed logistic regression model with LTCF as random factor and country as fixed factor. Lastly, to assess factors associated with medication prescription, a multilevel binary logistic regression model was built. All residents’ and LTCFs’ characteristics, showing a difference in opioid use prevalence with *P* < 0.100 in univariable multilevel analysis were included in a stepwise backward model building procedure, with *P* < 0.01 as boundary for statistical significance. Country was included as fixed effect to compare data between countries, LTCF was defined as a random effect. Other fixed effects were age category, gender, length of stay, BANS-S score, dementia status and cause of death on resident level and staffing structure on LTCF level. The resulting model was applied to explore associations with prevalence of antipsychotic and hypnotic use. Associated factors were calculated for the entire survey population and per country. The estimated variance between LTCFs was used to calculate the adjusted intraclass correlation coefficients on the LTCF level to explore variation between LTCFs.^13^ Statistical analyses were performed in SPSS 23.

## Results

The PACE database contains data from 1707 deceased residents in 322 LTCFs. The nurses response rate was 81.6% (ranging from 54.2% in England to 95.1% in Finland). For this survey, we excluded 323 residents of whom we did not receive the nurses’ questionnaire and 305 residents who died outside their LTCF, resulting in a study sample of 1079 residents, deceased in their LTCF.

### Residents’ and LTCFs’ characteristics

As shown in table 1, significant differences in LTCF type were identified. The deceased residents differed between countries by age, length of stay, BANS-S score, prevalence of dementia status and cancer versus non-cancer cause of death. Compared with other countries, LTCF residents were younger and had a shorter stay in Poland, where the cause of death was predominantly cardiovascular and cerebrovascular disease. Polish residents also had the highest BANS-S score, reflecting a higher dependency rate in daily life activities. Finnish LTCF residents had the highest percentage of dementia.

**Table 1:** Comparison of resident characteristics between countries

Country	Poland	Italy	Finland	England	Belgium	The Netherlands	*P* value
*n*	234	144	196	72	237	196	
LTCF type							
Type of LTCF where residents died							
Physicians and nurses offsite	0%	0%	0%	43.1%	0%	0%	<0.001^a^
Physicians off-site, nurses on site	32.5%	75.2%	100%	56.9%	100%	38.9%	
Physicians and nurses on site	67.5%	24.8%	0%	0%	0%	61.1%	
Resident characteristics							
Residents’ gender (% female)	65.5	66.7	68.6	74.6	63.8	67.0	0.668^b^
Residents’ age in years							
≥90	22.4%	27.8%	36.4%	46.0%	43.9%	40.9%	0.036^a^
80–89	46.1%	55.5%	52.3%	38.1%	45.7%	42.6%	
<80	31.5%	16.7%	11.3%	15.9%	10.4%	16.5%	
Residents’ mean age (SD)	81.3 (11.0)	85.6 (7.5)	86.6 (8.2)	88.3 (7.3)	87.5 (7.5)	86.9 (8.1)	<0.001^c^
Length of stay in years (SD)	1.8 (3.0)	2.3 (3.2)	2.7 (2.9)	2.5 (3.0)	3.4 (3.6)	2.9 (3.2)	0.002^c^
Mean total BANS-S score^d^ (SD)	22.4 (4.3)	21.9 (3.9)	20.0 (3.8)	17.7 (3.9)	19.1 (4.8)	18.2 (4.6)	<0.001^c^
Resident with dementia^e^	67.7%	79.1%	87.2%	60.3%	66.4%	65.9%	<0.001^b^
Cause of death							
Non-cancer	95.2%	89.8%	91.9%	80.0%	89.7%	91.1%	
Cancer	4.8%	10.2%	8.1%	20.0%	10.3%	8.9%	0.050^b^

a Calculated with multinomial logistic regression.

b Calculated with binary logistic regression.

c Calculated with linear regression.

d BANS-S. Seven-item scale, scores range 7–24, higher scores indicate higher functional disability and dependency.

e In this survey, a resident ‘with dementia’ is a resident which is designated as ‘suffering from dementia’ by the nurse and/or the physician.

### Medication prescription prevalence

**Figure 1:**
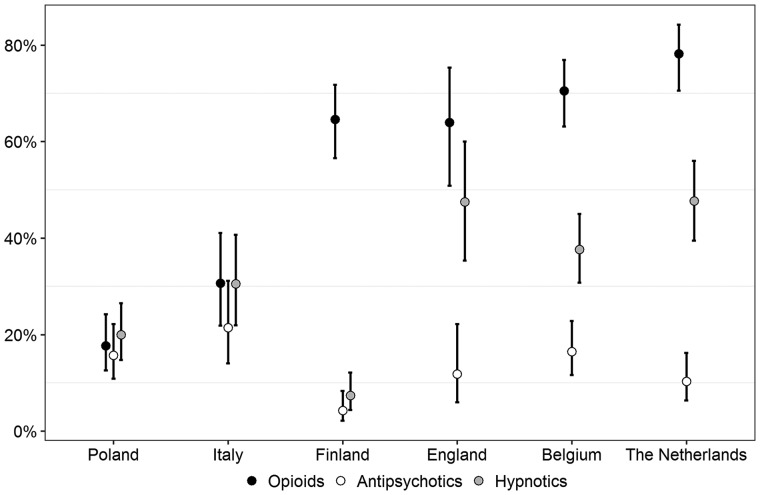
Estimated percentage of residents with opioid, antipsychotic and hypnotic prescription in last 3 days of life

The estimated prevalence of opioid, antipsychotic and hypnotic prescription in the last 3 days of life (figure 1) differed (*P* < 0.001) between countries. Opioid prescription varied from 18.5% in Poland to 77.9% in the Netherlands. Antipsychotic prescription varied from 4.8% in Finland to 22.4% in Italy. Hypnotic prescription ranged from 7.8% in Finland to 47.9% in the Netherlands.

### Factors associated with medication prescription

After statistical adjustment for LTCF type and resident’s characteristics, odds of opioid prescription in the last 3 days of life were significantly higher in all countries than in Poland, with exception of Italy (table 2). ORs ranged from 9.46 in Finland (95% CI: 4.58–19.52) to 23.11 in England (95% CI: 7.12–75.4 Opioid prescription was associated with the BANS-S score (OR 1.07; 95% CI: 1.03–1.11), reflecting an increase in opioid prescription for residents with more severe physical disability. Odds of opioid prescription was 3.5 times higher for residents dying of cancer (OR 3.51; 95% CI: 1.83–6.72) compared with residents dying of non-cancer causes.

**Table 2:** Resident and LTCF characteristics associated with medication prescription in the last 3 days of life

	Opioids	Antipsychotics	Hypnotics
	OR (95% CI)	OR (95% CI)	OR (95% CI)
Country			
The Netherlands	21.22 (10.37–43.39)	0.56 (0.27–1.19)	3.80 (1.98–7.30)
Belgium	14.68 (6.92–31.11)	0.79 (0.38–1.64)	1.71 (0.86–3.39)
England	23.11 (7.12–75.04)	0.78 (0.24–2.58)	3.42 (1.23–9.48)
Finland	9.46 (4.58–19.52)	0.23 (0.09–0.56)	0.24 (0.11–0.53)
Italy	1.76 (0.83–3.73)	1.22 (0.59–2.50)	1.14 (0.55–2.36)
Poland	Ref.	Ref.	Ref.
Type of LTCF			
Physicians and nurses off-site	0.37 (0.08–1.65)	1.40 (0.26–7.47)	1.10 (0.27–4.47)
Physicians off-site, nurses on site	0.99 (0.54–1.82)	1.24 (0.66–2.32)	1.87 (1.05–3.32)
Physicians and nurses on site	Ref.	Ref.	Ref.
Resident’s gender			
Female	1.10 (0.78–1.57)	0.85 (0.55–1.30)	0.93 (0.65–1.32)
Male	Ref.	Ref.	Ref.
Resident’s age in years			
≥90	1.00 (0.59–1.68)	0.76 (0.40–1.42)	0.56 (0.33–0.93)
80–89	1.12 (0.69–1.80)	1.20 (0.68–1.09)	0.93 (0.58–1.48)
<80	Ref.	Ref.	Ref.
Length of stay in years	0.94 (0.89–0.99)	1.000 (0.99–1.01)	0.94 (0.89–1.00)
Total BANS-S score^a^	1.07 (1.03–1.11)	0.98 (0.93–1.02)	0.99 (0.95–1.02)
Resident with dementia^b^			
Yes	0.96 (0.65–1.40)	0.96 (0.60–1.54)	0.84 (0.57–1.24)
No	Ref.	Ref.	Ref.
Cause of death			
Cancer	3.51 (1.83–6.72)	1.65 (0.86–3.13)	2.10 (1.20–3.67)
Non-cancer	Ref.	Ref.	Ref.

Multilevel multivariable logistic regression.

Opioids: 917 included, 162 missing. Antipsychotics: 917 included, 162 missing. Hypnotics: 908 included, 171 missing.

a BANS-S. Seven-item scale, scores range 7–24, higher scores indicate higher functional disability and dependency.

b In this survey, a resident ‘with dementia’ is a resident which is designated as ‘suffering from dementia’ by the nurse, the physician or both.

Odds of antipsychotics were about a fourth in Finland (OR 0.23; 95% CI: 0.09–0.56) in comparison to Poland.

Odds of hypnotic prescription were about a fourth in Finland (OR 0.24; 95% CI: 0.11–0.53) though were higher in the Netherlands (OR 3.80; 95% CI: 1.98–7.30) compared with Poland. Odds of hypnotic prescription were higher (OR 2.10; 95% CI: 1.20–3.67) for residents dying of cancer, than for residents dying of other causes. In this study, the dementia status did not show any significant association with opioid, antipsychotic and hypnotic prescription.

The intra-class correlation coefficient within the level of LTCFs was 14.7 for opioid prescription, meaning that about 15% of the variation was due to factors of the LTCF. The intra-class correlation coefficient within the level of the LTCF was 9% for antipsychotic and 13% for hypnotic prescription.

### Factors associated with medication prescription within a country

We found associations between opioid prescription and dying of cancer within some countries. Compared with residents, deceased of non-cancer diseases, odds for receiving opioids amongst residents dying of cancer were 14.28 in Italy (95% CI: 2.26–90.3) 8.96 in Poland (95% CI: 1.6–49.40; *P* = 0.012). We found no other significant associations for cancer. Other variables did not show significant associations with medication prescription in any of the countries studied, which could be explained by small sample sizes per country.

## Discussion

### Principal findings

In our study, we found significant differences between six European countries in opioid, psychotic and hypnotic prescriptions in the last 3 days of life of LTCF residents. The most striking differences were found in opioid prescription estimated percentages, ranging from 18.5% in Poland to 77.9% in the Netherlands. Low prevalence of antipsychotic (4.8%) and hypnotic (7.8%) prescription in Finland was also noteworthy. Differences in medication prescription between countries stood firm after multiple statistical adjustment, meaning that LTCF type or residents characteristics alone do not explain these differences. Country appears as the most important determinant for the prevalence of opioid, antipsychotic and hypnotic prescription in the last 3 days of life in LTCFs. Dying of cancer triples the odds of opioid prescription and doubles the odds of hypnotic prescription in LTCFs’ residents’ end of life.

### Relation to other studies and possible explanations

Opioid prescription prevalence in the last days of life of 60%^14^ and 70%^15^^,^^16^ is regularly documented. Klapwijk^17^ even described opioid prescription prevalence up to 100% of LTCF residents of whom death was expected. Also in our study, the Netherlands had the highest opioid prescription prevalence. The low prevalence of opioid prescription in Italy (31.7%) and Poland (19.6%) is remarkable, but consistent with other research. In both countries, opioid prescription per capita was found to be lower than in other West-European countries.^18^ Opioid prescription in LTCFs may reflect the low prevalence in both countries^7^^,^^19^ in general. Although death is not often easily predictable in LTCF residents, prevalence of pain increases to 78% and shortness of breath to 52% in the last week of life.^16^ Taken into account the high prevalence of pain in LTCF residents, low opioid prescription prevalence in Italy and Poland could be questioned, since guidelines consider opioids as a as an effective treatment for pain symptoms during the last days of life and also recommend opioids to treat dyspnoea in the last days of life.^10^^,^^20^

Low opioid availability and prescription in Poland was described in an EU report.^21^^,^^22^ The ATOME group identified legislative barriers to opioid consumption in Poland: palliative care support initiatives are almost exclusively consulted for cancer patients to whom every cancer treatment is declined due to the feeble prognostic. Moreover, complete reimbursement of opioids was exclusively entitled to cancer patients, whereas other patients receive only 30% reimbursement in Poland. The low frequency of medical consultations could be an additional factor. Visits of the treating physician are planned every fortnight or even once monthly. As symptom burden,^17^ and hence, the need for treatment^15^ quickly evolves in the last days of life, this frequency is too low to provide effective response. Popular convictions and attitudes are another barrier to effective opioid use. The ATOME report^22^ already defined fear of opioids as an important factor. This fear and negative image of opioids is wide spread in the Polish society, even amongst healthcare professionals. In Italy, the reluctance to communicate openly^23^ about end of life and dying could explain the low opioid prescription, since opioids, by mistake, still might be seen as life-shortening^23^ or causing respiratory depression.^24^ For the same reason, family members often oppose opioid use,^23^ although cancer seemed to be a potent driver of opioid prescription, as opposed to non-malignant conditions.

Antipsychotic prescription prevalence in LTCF end-of-life care is, in our findings, lower than published in other research,^15^ and was remarkably low in Finland, which is consistent with another study. In Finland, there is a trend to decrease central nervous agents in patients, especially with dementia, as described by Pitkala.^4^

The wide variation in hypnotic prescription prevalence between participating countries might reflect different clinical practices regarding hypnotic prescription.

In our study, we found no association between dementia and medication prescription. Poorer analgesic treatment for persons with dementia has been documented,^25^ but for opioid treatment, a shift in awareness has been noticed in the last decade.^26^^,^^27^ Our findings might be considered confirmatory of this trend, suggesting that residents with dementia are as likely as those without to receive opioids. Moreover, in Finland, the increase in opioid prescription is established concomitantly with the decrease in psychotropic medication.^4^

Residents dying of cancer are 3.5 times more likely to receive opioids, compared with residents who died of other diseases. Opioid treatment for non-malignant pain remains a controversial issue.^28^ Guidelines advise caregivers to remain vigilant about long-term side effects and opioid dependence.^29^ As a consequence, physicians and their patients feel insecure about misuse and addiction^30^ and tend to have a wait-and-see approach towards opioids. Physicians often doubt the objectivity of non-malignant pain complaints, the appropriateness of opioid therapy and feel less confident and more concerned to prescribe opioids for non-malignant pain, compared with cancer-related pain. They attribute their reluctance to a lack of knowledge and training.^31^ This may explain why the discrepancy in opioid prescription between cancer pain and non-malignant pain is more explicit in countries with lower palliative care knowledge.

### Strengths and limitations

To our knowledge, this is the first European cross-country study concerning opioid, antipsychotic and hypnotic prescription in the dying phase in LTCF residents. The large population sample, the cross-country survey design, the focus on all LTCF deaths, regardless of cause of death and the high response rate are the main strengths of this study. Nevertheless, some limitations have to be acknowledged. We excluded 305 residents who died outside the LTCF. Some of them were transferred to the hospital for further diagnosing, for symptom control, because an exacerbation of their general condition or because a life-threatening situation occurred. This exclusion may lead to a bias and underestimate the medication use in the dying phase. Second, the study only provided dichotomous data on medication prescription: whether or not, an opioid, antipsychotic or hypnotic was prescribed in the last three days of life. We had no information about the indication, the prescribed dosage nor the prescription date. Higher dose of opioid prescription is not an unambiguous proof of improved treatment since overuse or misuse of opioids cannot be excluded based on the data we have got available. The link between the residents’ symptom burden and drug prescription needs further consideration. Whether or not death was expected, is not taken into account in our study. Since this information was provided by the physicians’ questionnaire, with a lower response rate (68, 3%), we did not take it into consideration to avoid drop-out. Finally, although the response rate differs between countries, it was only the low response rate in England which hampers the generalisation in England. Furthermore, the non-respondents analysis for care staff showed no significant differences in the residents demographic and clinical characteristics and the stay in the nursing home between residents with and without participating nursing staff.

### Implications for clinicians and policymakers

Our study pointed to the existence of important differences in medication prescription in the last three days of life of LTCF residents between participating European countries. Further research is needed to explain these differences. Disseminating correct information on the indication of use to the broad public and developing palliative care knowledge amongst health care professionals remain important points of action.

The existing difference in medication prescription between cancer and non-cancer patients is a concern both for clinical practice and research. In palliative care, clinicians should focus on the symptoms as such, and aim to improve the patients comfort, regardless of the underlying cause. Symptom relieve is not justified because a patient has cancer, but because he or she is suffering. In any case, palliative guidelines need to be developed or refined for older patients and those dying from non-cancer diseases, taking the multi-morbidity, the specific responsiveness and vulnerability for side effects of older patients into account.

## Supplementary Material

Supplementary Appendix
