# Supplementary material for: Opioid, antipsychotic and hypnotic use in end of life in long-term care facilities in six European countries: results of PACE
Source: Eur J Public Health. 2018 Oct 4;29(1):74–9. doi: 10.1093/eurpub/cky196 (PMC6345144; doi:10.1093/eurpub/cky196)
Supplement: Supplementary Appendix [file cky196_appendix_1.docx]

**Appendix 1: list of questions of the nurses’ PACE-questionnaire, used in this paper**

Were any opioids (e.g. Morphine, Oxycodone, Hydromorphone, Fentanyl, Buprenorphine, Tramadol) prescribed in the last 3 days of life? *(if necessary, look up in resident’s file)*

Yes

No

Were any antipsychotics (e.g. Haloperidol, Risperidone, Olanzapine, Clotiapine) prescribed in the last 3 days of life? *(if necessary, look up in resident’s file)*

Yes

No

Were any hypnotics and/or sedatives (e.g. Midazolam, Oxazepam, Lorazepam, Lormetazepam, Zopiclone, Zolpidem, Zaleplon) prescribed in the last 3 days of life? *(if necessary, look up in resident’s file)*

Yes

No

Bedford Alzheimer Nursing Severity-Scale (BANS-S) Please choose one item for each of the following 7 functions, indicating the condition of the resident 1 month before death.

a. Dressing

Usually is independent

Requires minimal assistance

Requires moderate assistance but is not totally dependent

Totally dependent

b. Sleeping

Usually has a regular sleep-wake cycle

Sometimes has an irregular sleep-wake cycle

Frequently exhibits irregular sleep-wake cycle

Severely disrupted sleep-wake cycle

c. Speech

Completely intact ability to speak

Somewhat decreased ability to speak

Moderately decreased ability to speak

Totally mute

d. Eating

Eats independently

Requires minimal assistance and/or coaxing

Requires moderate assistance and/or coaxing

Completely dependent

e. Mobility

Always able to walk independently

Sometimes able to walk independently

Able to walk only with help

Unable to walk even with help

f. Muscles

Very flexible and has full joint motion

Somewhat flexible with some joint motion impairment

Somewhat rigid

Contracted

g. Eye contact

Eye contact is maintained

Eye contact is usually maintained

Eye contact is rarely maintained

Never maintains eye contact

In your opinion, did the resident have dementia?

Yes

No

What was the place of death of the resident?

long term care facility

hospital

hospice/palliative care unit

other, please specify:

What was the underlying cause of death as far as you know?

cancer

cardiovascular disorder (not CVA)

cerebrovascular accident (CVA)/stroke

neurological disease (not dementia)

dementia

respiratory disease

other, please specify:
